# The Disappearing Seasonality of Autism Conceptions in California

**DOI:** 10.1371/journal.pone.0041265

**Published:** 2012-07-30

**Authors:** Soumya Mazumdar, Ka-Yuet Liu, Ezra Susser, Peter Bearman

**Affiliations:** 1 Paul F. Lazarsfeld Center for the Social Sciences, Columbia University, New York, New York, United States of America; 2 New York State Psychiatric Institute, Mailman School of Public Health, Columbia University, New York, New York, United States of America; University of Jaén, Spain

## Abstract

**Background:**

Autism incidence and prevalence have increased dramatically in the last two decades. The autism caseload in California increased 600% between 1992 and 2006, yet there is little consensus as to the cause. Studying the seasonality of conceptions of children later diagnosed with autism may yield clues to potential etiological drivers.

**Objective:**

To assess if the conceptions of children later diagnosed with autism cluster temporally in a systematic manner and whether any pattern of temporal clustering persists over time.

**Method:**

We searched for seasonality in conceptions of children later diagnosed with autism by applying a one-dimensional scan statistic with adaptive temporal windows on case and control population data from California for 1992 through 2000. We tested for potential confounding effects from known risk factors using logistic regression models.

**Results:**

There is a consistent but decreasing seasonal pattern in the risk of conceiving a child later diagnosed with autism in November for the first half of the study period. Temporal clustering of autism conceptions is not an artifact of composition with respect to known risk factors for autism such as socio-economic status.

**Conclusion:**

There is some evidence of seasonality in the risk of conceiving a child later diagnosed with autism. Searches for environmental factors related to autism should allow for the possibility of risk factors or etiological drivers that are seasonally patterned and that appear and remain salient for a discrete number of years.

## Introduction

Investigating seasonal patterns of births and conceptions of children with neurodevelopmental disorders can help identify underlying etiological drivers [1]. In this article, we investigate the seasonality of autism, a neurodevelopmental condition with rising prevalence over the past two decades [2]. While a large number of possible etiological drivers of autism have been suggested [3], there is little consensus as to its cause.

**Figure 1:**
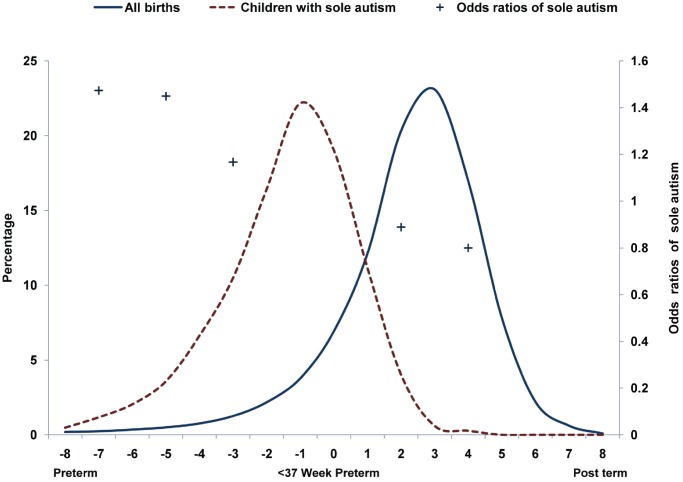
Distributions of gestational ages of children with autism and of all births and the odds ratios of sole autism by gestational age.

Previous studies of seasonality of psychiatric conditions, including schizophrenia, epilepsy, language disorders [1], [4]–[11], and especially autism, suffer from a number of shortcomings. First, studies of the seasonality of autism, with one exception [12], analyze the risk of autism associated with the timing of birth [5], [6], [8], [9], [13]. While this design is reasonable for disorders caused by exposure to seasonally patterned, peri-natal or post-natal exposures, it is unsuitable if the most crucial exposures are prenatal, as has been suggested in the case of autism [3]. Seasonality of births is particularly uninformative of prenatal exposures if preterm birth is common among the case population, as is true of children with autism [7]. Using data from California (described in detail in the methods section), Figure 1 compares the distribution of births of children with autism with that of all births as a function of gestational age. Approximately 11% of children with autism conceived between 1992 and 2000 were born at least three weeks preterm (less than 37 weeks gestation) as opposed to 1.3% of all children born in the same cohorts. Consequently, focusing on conception rather than on birth is preferable for the study of seasonality and autism risk. Second, most prior studies rely on chi-square tests, which necessitate the aggregation of data to arbitrary units of time [7], [8]. Similarly, many studies have examined the seasonality of autism births using data aggregated to (or stratified by) calendar units, such as seasons or months [5], [7], [8], [12], [14], [15]. However, these are not necessarily the units at which seasonality manifests [4], [7], [8]. Aggregating to conventional time units, while convenient, is methodologically problematic. Such aggregation decreases statistical power, sensitivity, specificity and increases the number of false positives and false negatives across spatio-temporal domains [16], [17]. Therefore, designs that are free of arbitrary calendar units are more reliable than those that depend on such aggregation. Third, many studies of seasonality suffer from small sample size, making adjustment for known risk factors difficult [2], [8], [9]. Finally, many studies aggregate data across cohorts [7], [8]. This practice may conceal local variations in the data and/or reveal spurious ones [18]. For example, if an environmental driver were to appear in some years and not in others, the process of aggregation may mask and/or dilute its effect. In addition, even if an increased risk was detected in an aggregated dataset, it would subsequently be impossible to identify the years in which the driver appeared.

This study aims to address the methodological problems discussed above. We study risk of autism based on time of *conception* using a relatively new, flexible, yet statistically rigorous method – the one dimensional scan statistic [19]. We attempt to minimize data aggregation across the time series. The one dimensional scan statistic methodology does not constrain the data into pre-aggregated units of time such as months, weeks or seasons, does not impose any specific shape or structure to the risk function [20], and yet performs a maximum likelihood based hypothesis test for statistical significance. The dataset we use is a large, population based administrative dataset consisting of all births and most autism cases in California. We explore the results for a number of compositional artifacts including parental age and socio-economic status (SES). We then discuss the relevance of our findings in the context of possible etiological drivers.

## Methods

### The Dataset

Case data were obtained from the California Department of Developmental Services (DDS). In California, children with Autistic Disorder (DSM IV code 299.0) are eligible for services from the DDS. Children with other conditions on the autism spectrum, such as Asperger’s and Pervasive Developmental Disorder-Not Otherwise Specified, are ineligible for DDS services. All autism diagnoses are confirmed by DDS staff, either by on-site diagnostic teams or by professionals with expertise in diagnosing autism. Diagnoses may be based on one or more of several diagnostic instruments, including the Autism Diagnostic Interview [21] or the Autism Diagnostic Observation Schedule [22]. To obtain information on prenatal, birth and parental characteristics, the DDS data were linked to the California Birth Master Files (BMF) for all children born from 1992 to 2005 using Link King software [23]. Links were based on first, middle, and last names, sex, race, date of birth, and maternal zip code at birth. Using these variables, links were assigned probabilistic matching scores. All links with match scores below 80% were manually reviewed. About 80% of children in the DDS database were linked to a birth record [24]. Children whose DDS records were not linked to a birth record are likely to have been born outside of California [25]. A more detailed description of the data linkage process can be found in our previous studies, which have used this linked dataset extensively [18], [24], [26]–[30].

Children with autism not co-morbid with mental retardation, or ‘‘sole autism,’’ were considered cases in the following analyses, and all other live births in the BMF were used as population controls. The BMF includes a number of covariates, including child’s sex, parents’ ages, parents’ education levels, and prenatal risk factors. The BMF also contains the date of the mother’s last menses to which we added a fortnight to obtain an approximate date of conception. Births paid for by Medi-Cal, California’s Medicaid program, were coded as 1, else 0. Preterm births (gestational age <37 weeks) were coded as 1. It is standard practice to categorize children born at less than 37 weeks gestation as preterm [31]–[33]. Low birth weight was coded as 1 for neonates weighing less than 2.5 kilograms. We define mean parental age as the mean of the mother’s age and father’s age or only the mother’s age when the father’s age was missing. For each cohort, approximately 9% of the records were missing information on father’s age. While it may be possible to examine maternal age alone effectively, given the conflicting evidence as to which combination of maternal and paternal ages is most strongly associated with autism diagnosis [25], [34], mean parental age is also an appropriate measure. We coded mean parental ages of 35 and above as 1, choosing this cutoff point based on existing literature [25], [34]. A negligible number of records (<1%) missing both mother’s and father’s ages were dropped from the dataset. The same process used to calculate mean parental age was used to calculate mean parental education, which was then categorized as <12 years, 12–16 years or >16 years to coincide with the approximate number of years of statutory education. Race/ethnicity was classified into five categories: non-Hispanic white, Hispanic white, Black, Asian, and other.

In the late 1990’s and early 2000’s, most autism diagnoses were made by age 6 years. Therefore, in order to ensure complete ascertainment of autism, we limited our sample to children who were conceived between 1992 and early 2000. The final dataset has 8,074 children with sole autism and 3,888,495 births as population controls. Our dataset is representative of children with autism in California, since it is estimated that the DDS serves the vast majority of this population [35]. Table 1 provides descriptive statistics of the study population.

**Table 1:** Descriptive statistics on the study population, N (%)^a^.

Calendar Year	Live births	Autism Cases	Medi-Cal	Preterm	Male	Low BirthWeight	Mean ParentalAge ≥35	Mean Parental Education (years)		Race/ethnicity			
								12–16	16+	Hispanic white	Black	Asian	Other
1992	525,795	310	223,996	52,669	269,590	28,672	88,431	194,438	42,696	222,905	38,965	46,294	11,756
			(43)	(10)	(51)	(5)	(17)	(37)	(8)	(42)	(7)	(9)	(2)
1993	511,918	488	222,976	51,454	262,343	28,053	90,128	149,362	39,257	229,030	37,612	50,028	8,519
			(44)	(10)	(51)	(5)	(18)	(29)	(8)	(45)	(7)	(10)	(2)
1994	477,627	728	208,886	29,290	243,624	19,476	86,980	140,545	37,378	215,463	33,204	48,093	8,761
			(44)	(6)	(51)	(4)	(18)	(29)	(8)	(45)	(7)	(10)	(2)
1995	488,756	1,032	209,947	37,446	250,106	22,010	93,116	143,299	39,819	225,995	32,890	50,519	8,597
			(43)	(8)	(51)	(5)	(19)	(29)	(8)	(46)	(7)	(10)	(2)
1996	483,284	1,180	197,673	28,938	247,184	26,474	96,943	176,630	31,014	225,871	32,897	50,520	9,534
			(41)	(6)	(51)	(5)	(20)	(37)	(6)	(47)	(7)	(10)	(2)
1997	461,626	1,306	176,503	18,061	235,835	25,491	95,510	183,970	29,407	215,381	30,952	48,618	10,308
			(38)	(4)	(51)	(6)	(21)	(40)	(6)	(47)	(7)	(11)	(2)
1998	407,297	1,280	151,391	14,311	243,341	20,845	86,090	164,527	25,766	190,659	26,720	43,305	9,091
			(37)	(4)	(52)	(5)	(21)	(40)	(6)	(47)	(7)	(11)	(2)
1999	405,513	1,311	151,165	13,863	221,161	20,784	88,800	167,410	26,892	192,617	25,241	45,975	9,118
			(37)	(3)	(52)	(5)	(22)	(41)	(7)	(47)	(6)	(11)	(2)
2000^b^	126,679	439	49,276	8,816	65,217	10,309	28,269	51,319	8,509	61,130	8,212	15,692	2,572
			(39)	(7)	(51)	(8)	(22)	(41)	(7)	(48)	(6)	(12)	(2)

a Percentages are relative to all live births. Categories not shown (<12 years of education and Non-Hispanic white) are the reference categories in our analyses. Their comparable statistics can be calculated easily from the table.

b Data for the year 2000 (shown here and as used in the analyses) consist of children conceived in January through March.

We also used the BMF to create a dataset of siblings who were conceived between 1991 and 2005 in order to examine whether families at increased risk for having a child with autism exhibit distinct family planning behaviors that could manifest as temporal clusters. We refer to this as the “assortative conception hypothesis.” Siblings were determined by exact matching on mother’s date of birth, the first letter of mother’s maiden name and father’s date of birth. The siblings’ dates of conception were calculated using the same method as described above for the BMF. We have used this dataset extensively in previous research [24], [27], [36], and it is comprised of approximately 4,800,000 children.

Finally, we used publicly available influenza data to explore whether the temporal patterning of influenza could be associated with excess temporal risk of autism. We have data reporting influenza mortality for California from 1994 to 1998 [37] and influenza incidence from 1999 onwards [38].

### Methods

In this paper, the term “temporal cluster” refers to an excess of conceptions of children later diagnosed with autism relative to the expected number of conceptions of children later diagnosed with autism in a given period of time. Searching for excess risk, periodic or not, is thus equivalent to searching for clusters in a time series. If clusters are found in the same period of time, year after year, it would suggest periodicity or seasonality. To conduct this analysis, we used a one-dimensional Bernoulli scan statistic that is widely used to assess the spatial, temporal and spatio-temporal clustering of events. The one-dimensional scan statistic [19], [39] reduces the problem of detecting an excess number of events over a global time series to a single maximum likelihood based hypothesis test. Once found, the value of the likelihood ratio test statistic for the “most likely” cluster then needs to be compared to a reference distribution, which cannot be theoretically derived. A Monte Carlo simulation [40], also known as random permutation testing, is used instead. The scanning procedure and Monte Carlo simulation are described in detail in the supplementary material (Appendix S1).

Since the Monte Carlo testing procedure is dependent on the total number of events in the time series n_G_, it is insensitive to an increasing trend within the time series. However, this problem is addressed by dividing the time series into sections and carrying out separate tests in each section with appropriate adjustment for multiple testing described below. Since our data exhibit an increasing trend, the total number of cases and their corresponding rates will have a greater value of n_G_ in the later sections than in earlier years. Note that the adjustment for an increasing trend improves with smaller section size, but smaller sections also increase the number of independent hypothesis tests and decrease statistical power.

We divide our time series into six overlapping sections or windows ***W***, each of size three years. We also experimented with windows of four year size. There was no difference in the timing of the clusters that were found, but the corresponding relative risks decreased. This window ***W*** should not be confused with the windows or zones used in the maximum likelihood maximization procedure. To correct for multiple testing, we use a conservative Holm-Bonferroni correction [22]. In addition, it is necessary to choose a consistent cutpoint at which the time series is divided into sections. We use March as the start and end points of ***W***, since, given a prenatal exposure hypothesis, March is the least likely month in which an excess risk of autism conceptions should be observed [5], [7]. The results were also robust to shifting the start/end period from March to a different month, such as December. Some researchers prefer to decompose a time series based on trend and seasonality. Others consider explicit de-trending of a time series essential before any analysis. De-trending a time series is meaningful in analyses when there is sufficient knowledge about the drivers of the trend. However, if multiple etiological agents are contributing to an observed trend, it is likely that the nature of nonstationarity in the time series is complex. In the case of autism, the increasing trend is likely a result of a complex interplay of social and environmental process [24]. Assuming arbitrary trends and removing them could lead to spurious periodicity [41] and would dramatically decrease statistical power. It is also important to note the difference between multiple calculations (optimization) done on data in order to maximize the likelihood of an estimator *before and in order to test a hypothesis*, as opposed to testing multiple hypotheses on different aggregations of the same dataset. The former approach is an accepted means of statistical parameter estimation [42] and is the procedure used by the spatial scan statistic. The parameter that is estimated is the size and location of the most likely cluster. This is different from multiple testing which consists of multiple hypothesis tests on a single dataset without appropriate corrections for multiple testing. Our method conducts a single maximum likelihood hypothesis test on each window ***W.***

In the study of the seasonality of disorders, competing approaches to modeling time series data include fitting pre-specified designs, such as splines [20], and mapping periodicity and seasonality with either a Fourier series or Wavelets [43]. Unlike these methods, our approach does not require the data to have shapes and/or periodicities. Another approach is to use empirical mode decomposition [9], which does not impose a structure to the data, but usually does not provide a measure of statistical significance.

**Figure 2:**
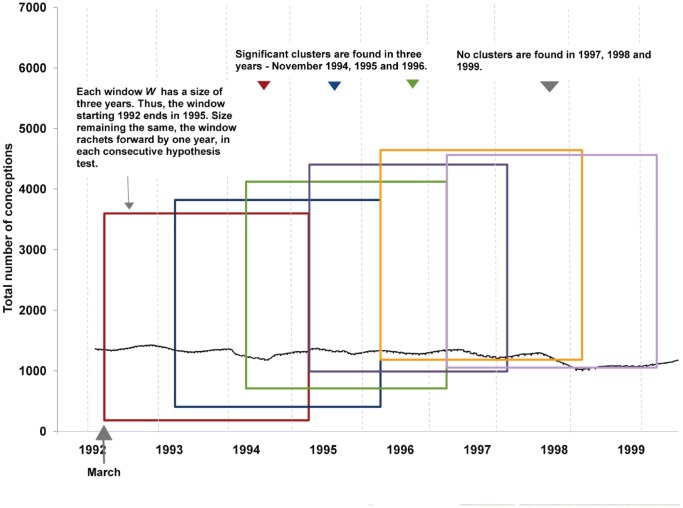
Scanning method and results. This figure is illustrative; for the precise time periods of the clusters and relative risks, see Table 2.

The scanning procedure is summarized in Figure 2. The entire analysis was accomplished with the SaTScan software, which is a freeware program that realizes the scan statistic and carries out the Monte Carlo hypothesis testing described above [44].

**Table 2:** Temporal clusters of conceptions of children later diagnosed with autism from three year scans.

Temporal window*W*	Temporal cluster ofsole autismconceptions	Total number ofconceptions intemporal cluster	Number of cases intemporal cluster	Expectednumber ofcases	Relative Risk	*P*-value
1992/3/1 - 1995/3/1	1994/11/7 - 1994/12/2	35,323	82	39.93	2.11	0.0001
1993/3/1 - 1996/3/1	1995/11/9 - 1995/12/7	38,602	109	62.91	1.72	0.0003
1994/3/1 - 1997/3/1	1996/11/7 - 1996/12/6	41,615	134	88.75	1.53	0.0103

### Robustness Checks and Other Tests

It is pssible that observed temporal clusters of conceptions would be due to compositional differences with respect to autism risk factors [2], [45]. For example, since male sex is an established risk factor for autism [45], a spurious temporal cluster could be observed due to an increased number of conceptions of males in a given period. To rule out confounding effects, we predict cluster status as a function of known autism risk factors using logistic regression models within each time period ***W*** in which clusters were found. Specifically, separate regressions are conducted for the periods 1992/3/1 to 1995/3/1, 1993/3/1 to 1996/3/1 and 1994/3/1 to 1997/3/1. For each of these periods, days that belonged to the cluster are coded as one and days outside of the cluster as zero. Cluster status is predicted as a function of sex, whether mean parental age is greater than 34, parental education, race/ethnicity and insurance status. Separately, we regress preterm birth and low birth weight as functions of cluster status. Note that these covariates are exactly as described earlier and summarized in Table 1.

We also test the assortative conception hypothesis, the theory that parents at greater risk of having children with autism may time conception differently than those at lesser risk, which would manifest in temporally consistent family planning behaviors. Such a birth pattern was observed with schizophrenia [46], for which it was suggested that parents at higher risk of having children with schizophrenia plan summer deliveries and, therefore, conceive in the autumn. To assess whether such dynamics are at play in the case of autism, we use the sibling dataset. We calculate the percentage of siblings of cases and of controls in the cluster period who, independent of the year in which they were conceived, were conceived within a time window *t* of each other. We test timeframes of 15, 22.5, 30 and 45 days. If the assortative conception hypothesis is true, then a significantly greater percentage of case siblings would have been born in shorter timeframes than control siblings. Note that, while the dates of conception are assumed to be 15 days after the date of last menses, it is possible that some children were actually conceived a few days earlier or later. Therefore, the 15 day timeframe may miscategorize some sibling pairs. This is not an issue for the longer timeframes, since they are able to capture siblings whose dates of conception were misestimated by a few days.

In addition, we explore whether the temporal patterning of influenza could be associated with excess temporal risk of autism. Given that very limited flu exposure data at the state and county levels to the resolution of a day are available from public sources, we can only make tentative conclusions based on exploratory analyses of the extant ecological data.

Finally, we examine whether patterns of seasonality can be localized to a specific geography. We segment our data in two ways: by metropolitan and adjacent-to-metropolitan residence based on Rural-Urban Commuting Areas codes available from the Rural Health Research Center at the University of Washington and by North and South California along the northern boundaries of San Luis Obispo, Kern and San Bernardino Counties. We then repeat our scanning procedure on each geographic segment.

This study was approved by the Columbia University Institutional Review Board and by California’s Committee for the Protection of Human Subjects.

## Results

Table 2 lists the periods when temporal clusters of conceptions of children later diagnosed with autism occurred along with their relative risks. Figure 2 graphically shows the cluster periods as well as the scan procedure and the result, however the figure is meant to be illustrative. Our methodology allowed us to pin point the exact dates of excess risk instead of vague indications of risk by months and seasons. Specifically, we observe a significant increased risk (RR >1.5, p < 0.05) of sole autism among children conceived in the last three weeks of November and the first week of December for the years 1994, 1995 and 1996 (Table 2). The relative risks declined from the beginning of our study period and became insignificant; no significant clusters were detected in 1992–1993, 1997, 1998 or 1999.

Tests for confounding by autism risk factors using logistic regression models for each time period ***W*** show no consistent trends in the composition of the temporal clusters (Table 3). The three windows or time periods are 1992/3/1 to 1995/3/1, 1993/3/1 to 1996/3/1 and 1994/3/1 to 1997/3/1. For example, while children of parents with 12 to 16 years of education are under-represented in the 1994 cluster (OR 0.95, p<0.05) relative to children of parents with high school educations, they are over-represented in the 1996 cluster (OR 1.40, p<0.05). Relationships between key autism risk factors and having been conceived during a cluster period are neither consistently significant nor unidirectional.

**Table 3:** Odds ratios of being conceived within a temporal cluster.^c^.

	Conceived in the 1994 cluster			Conceived in the 1995 cluster			Conceived in the 1996 cluster		
	Odds ratio	CI	*P*-value	Odds ratio	CI	*P*-value	Odds ratio	CI	*P*-value
Male	0.99	0.97, 1.01	0.26	1.00	0.99, 1.01	0.73	0.99	0.98, 1.00	0.30
Mean Parental Age ≥35	**1.06**	1.03, 1.09	<0.05	0.99	0.98, 1.00	0.53	**1.07**	1.06, 1.08	<0.05
Mean Parental Education (years)									
12 to 16	**0.95**	0.93, 0.98	<0.05	1.00	0.99, 1.01	0.98	**1.40**	1.38, 1.42	<0.05
16+	**0.93**	0.89, 0.98	<0.05	0.97	0.95, 0.99	0.10	**0.86**	0.84, 0.88	<0.05
Less than 12	Reference								
Race/ethnicity									
Hispanic white	1.02	1.00, 1.05	0.07	**0.96**	0.95, 0.97	<0.05	**1.19**	1.18, 1.20	<0.05
Black	**0.94**	0.90, 0.99	<0.05	**0.91**	0.89, 0.93	<0.05	1.04	1.02, 1.06	0.09
Asian	0.98	0.95, 1.02	0.35	**0.90**	0.89, 0.92	<0.05	1.01	0.99, 1.02	0.71
Other	0.94	0.86, 1.02	0.12	**0.91**	0.88, 0.95	<0.05	**1.29**	1.25, 1.34	<0.05
Non-Hispanic white	Reference								
Medi-Cal	0.98	0.96, 1.00	0.11	1.00	0.99, 1.01	0.99	**0.94**	0.93, 0.95	<0.05

CI: 95% Confidence Intervals.

c Odds ratios are relative to all conceptions within the time period window ***W*** in which the cluster is found. For example, odds ratios for children conceived in the 1994 cluster are relative to those conceived in the window ***W*** 1992/3/1 to 1995/3/1. Significant results are in bold.

Table 4 displays the odds ratios of a preterm or low birth weight (<2.5 kg) birth relative to normal term and normal birth weight for those conceived in each cluster relative to those conceived within each cluster’s respective time period ***W***. Preterm birth and low birth weight births show inconsistent and small associations with the observed temporal clusters. While the odds ratio of having been born preterm is 0.96 (p<0.05) for those conceived in the 1994 cluster relative to those conceived outside of the cluster but during the same time period, the odds ratio of having been born preterm is 1.03 (p<0.05) for those conceived in the 1995 cluster.

**Table 4:** Odds ratios of being A) born preterm and B) born with a low birth weight for those conceived within a temporal cluster.^d^.

Conception cluster	A) Preterm Birth			B) Low Birth Weight		
	Odds ratio	CI	*P*-value	Odds ratio	CI	*P*-value
1994	**0.96**	0.95, 0.96	<0.05	**0.98**	0.98, 0.98	<0.05
1995	**1.03**	1.02, 1.03	<0.05	**1.01**	1.01, 1.01	<0.05
1996	**0.98**	0.97, 0.98	<0.05	**1.00**	1.00, 1.00	<0.05

CI: 95% Confidence Interval.

d Odds ratios are relative to conceptions outside of the cluster but within the time period window ***W*** in which the cluster is found. For example, the reference category for those in the 1994 conception cluster is those who were conceived outside of the cluster but within window ***W*** 1992/3/1 to 1995/3/1. Significant results are in bold.

Table 5 displays the percentages of siblings of cases and of controls conceived within 15, 22.5, 30 and 45 days of their case and control siblings respectively, independent of year. There are no significant differences between cases and controls in any of the timeframes. There is thus no evidence that parents of children with autism are more likely to conceive assortatively than other parents; our findings do not support the assortative conception hypothesis.

**Table 5:** Percentages of siblings of cases and of controls conceived within timeframes of *t* days of conception of the respective case or control child, independent of the year of conception.

Timeframe (days)	Case percent	Control percent	*P*-value (χ^2^)
15	17.92	14.19	0.63
22.5	21.39	18.78	1.00
30	24.86	22.59	1.00
45	28.32	30.04	1.00

Exploratory analyses of flu mortality and incidence data revealed that the flu season in California generally peaks in the first two weeks of January. This trend was consistent throughout the entire study period. For example, approximately 75% of the reported flu cases of the 1993–1994 flu season occurred during these two weeks. Although these results should be interpreted with caution due to the possibility of ecological fallacy, they suggest that any etiological association of influenza exposure to autism within the observed clusters would have occurred at the end of the first month of pregnancy.

Geographically segmenting the data resulted in uneven numbers of conceptions in each of the datasets, making it hard to draw definitive conclusions. For example, approximately one third of all cases of autism are within Los Angeles. However, results from the scanning procedures for the Metro areas and for Southern California were most similar to the state-wide results described in Table 2, indicating a possible but not definitive ecological association with autism specific to these (or similar) areas.

## Discussion

We observe three clusters of conceptions of children later diagnosed with autism at exactly the same time of year for three consecutive years: 1994, 1995 and 1996. We do not detect any clusters in 1992–1993 or 1997–1999. We posit that an unknown etiological driver (or a combination of multiple drivers) caused the observed seasonal patterning of risk from 1994 to 1996. It is difficult to know whether this driver was present in 1992–1993, since the numbers of cases in these years are exceedingly small, averaging approximately 30 to 40 cases in a 30 day period. Thus, even if clusters (and thereby the driver(s)) were present, they may not have been detected due to lack of statistical power. In contrast, for the later period with no clusters, 1997–1999, the numbers of cases are sufficiently large (Table 1). The absence of seasonality during this period may be due to the disappearance of the etiological driver(s) or due to the introduction of other etiological drivers in other parts of the year that prevent the detection of a cluster.

What etiological drivers could be implicated by the observed seasonal patterning of conceptions of children later diagnosed with autism? Prenatal exposure to various risk factors or the absence of protective factors during specific gestational periods could explain some of the pattern we observe. However, since a specific exposure has not been established, we cannot estimate an exact timeframe within gestation. It is generally established that exposures during the embryonic stage, which consists of the first two trimesters of gestation, cause increased postnatal risk of neurodevelopmental and psychiatric conditions, including autism [47]. These exposures could be seasonally patterned, and a number of such exposures have been hypothesized [5].

Exposure to a certain strain of flu during the cluster years could explain the presence of clusters in some years and not in others. Exposure to flu during gestation has been associated with increased risk of schizophrenia in ecological studies [48]. However, the critical timing of such an exposure is somewhat uncertain. Only one study has related serologic measures of maternal influenza to schizophrenia. This study found that the largest association occurred during early gestation [49]. For mothers who conceive in November, the first trimester coincides with the California flu season [50]. Our analyses did not detect a difference in gross temporality of the flu season for the cluster versus non-cluster years. However, detailed analyses at the individual level that incorporate the specific virus type and blood viral load through serum antibody analysis could better address such hypotheses [49].

Negative deficit schizophrenia, the presentation of which is similar to autism, has been found to have an excess of July births [51]. This form of schizophrenia has been linked to the Borna virus, which could be infectious or activated seasonally. For autism, an association with the Borna virus is supported by animal models but not by human testing [52]. Links between maternal viral exposure and autism in children are an active area of study [52].

Meanwhile, various studies have found an excess of births of children later diagnosed with autism in the summer [1], [4], [9]. While children with autism are significantly more likely to be born preterm, we would nevertheless expect an excess risk of conceiving a child later diagnosed with autism in November to result in an excess of births of children later diagnosed with autism in the summer. For children conceived in November, the second trimester coincides with the pollen season [53], and in Northern California, maternal asthma in the second trimester is associated with an increased risk of autism [54]. Pollen seasons vary in intensity from season to season and year to year, which could be in accordance with the observed disappearance of seasonality.

Some scholars propose that a prenatal lack of vitamin D increases the risk of autism and other neurological deficits [55]. In humans, most required vitamin D is synthesized in the skin from sunlight, and much of our study area has insolation above the national average. However, vitamin D production is mediated by a number of other factors, including age, skin tone, individual behavior and pollution [56], [57]. Pollution is exacerbated in the winter in the Los Angeles metro area, where a large proportion of our cases were born [58]. Furthermore, our analyses of geographically segmented data support the possibility that the observed temporal patterning was driven by those in Southern California, metro areas. Therefore, environmental pollution, in combination with local meteorological conditions varying in certain years, could be a plausible driver of the observed seasonality pattern.

Our observations are consistent with the above theories, and any number of the etiological agents mentioned could be contributing to the observed clusters. It is important to note that the driver(s) of seasonality is not likely a critical driver of increased prevalence, even if it plays a modest role in increasing incidence, since autism prevalence keeps rising in the years in which seasonality was not detected. The identification of temporal clusters for autism can complement other findings in molecular biology and epidemiology, point to new mechanisms for greater study, and reject competing explanations. Note that theories suggesting that parents of children with autism select different months in which to have children are not supported by the results reported here. In contrast, the identification of seasonality may provide some support for an etiological role of influenza in the first trimester and/or of increased asthma in the second trimester of pregnancy. Studies from other jurisdictions using comparable definitions of autism and comparable methods need to be conducted in order to identify whether the patterns observed in California are unique.

The fact that our study utilizes data from California limits the generalizability of our results. In addition, we are unable to examine seasonality patterns beyond 2000 with the present dataset. Although our approach does not explicitly correct for increasing autism prevalence, our clusters are not due to this trend, since they are only present in the first half of the study period. Still, our use of a scan statistic for a disorder with increasing prevalence is of a slightly experimental nature, since it is not a common practice. While the DDS serves the vast majority of children with autism in California, it is not possible to determine whether children with autism who do not utilize DDS services or those who were not successfully matched to the BMF display a different pattern of seasonality.

### Conclusion

There is some evidence of seasonality in the timing of conceptions of children later diagnosed with autism. Significant excesses of risk are found in November in the years 1994, 1995 and 1996. These significant excesses of risk are not explained by known autism risk factors, such as parental education, age, socio-economic status or child’s sex, nor are they artifacts of planned conceptions by parents. The pattern of increased risk decreases from 1994 to 1996, and is not found in 1997, 1998 or 1999. Searches for environmental drivers of autism should, therefore, allow for the possibility of the existence of seasonally patterned yet temporally anisotropic risk factors.

## Supporting Information

Appendix S1 **The one dimensional scan statistic:** Scanning procedure and Monte Carlo testing
(DOC)
