# Supplementary material for: The Disappearing Seasonality of Autism Conceptions in California
Source: PLoS One. 2012 Jul 30;7(7):e41265. doi: 10.1371/journal.pone.0041265 (PMC3408493; doi:10.1371/journal.pone.0041265)
Supplement: Appendix S1 — The one dimensional scan statistic: Scanning procedure and Monte Carlo testing (DOC) [file pone.0041265.s001.doc]

Supplementary Material: The one dimensional scan statistic

The following description of the scan statistic is based on Kulldorff , who discusses the maximum likelihood based hypothesis testing procedure in the spatial context. The description has been adapted to the temporal situation.

N denotes a point process where N(A) is the random number of points in set A⊂ G, where G is a time series. A window moves over the time series, defined as a collection **Z** of purely temporal zones Z⊂ G. For the Bernoulli model, μ is a measure such that μ(A) is an integer for all subsets A⊂ G. Note that in probability theory a “measure” is a real-valued function defined as a set of events in a probability space that satisfies measure properties such as countable additivity . Each unit of measure corresponds to an individual who is either in a 1 (case) or 0 (population/control) state. Individuals in these states can be treated as points, and the locations of these individuals within the time series can be treated as a point process.

In the model, there is exactly one zone in the time series A⊂ G such that each individual within the zone has a probability p of being a point, while the probability of individuals outside the zone is q. The probability of any one point is independent of all others. The null hypothesis is H_0_: p=q. The alternative hypothesis is H_1_: p>q, Z∈ **Z.** Under H_0_, N(A) ~ Binomial(μ(A),p) for all sets A⊂ Z, and N(A) ~ Binomial(μ(A),q) for all sets not in Z, that is A⊂ Z^C^ . The choice of window that corresponds to the collection **Z** in the purely temporal case consists of all possible linear/temporal windows. If n_z_ denotes the total number of points or individuals in zone z, and n_G_ denotes the total number of observed individuals or points, then the likelihood function of the Bernoulli model is expressed as:

L(Z,p,q) = (p) ^n^z *(1-p) ^(μ(z)-n^z) * q ^(n^G ^- n^z) * (1-q) ^((μ(G) - μ(Z)) – (n^G ^-n^z)) (1)

The zone $\hat{\text{Z}}$ on the time series that maximizes the above likelihood function is most likely to be a cluster on the time series. In other words, $\hat{\text{Z}}$ is the maximum likelihood estimator of the parameter Z. Kulldorff shows the following procedure to calculate the maximum likelihood function conditioned on Z:

L(Z) is equal by definition to

sup L(Z,p,q) = (R) ^n^z *(1-R) ^(μ(z)-n^z) * R_c_ ^(n^G ^- n^z) * (1- R_c_) ^((μ(G) - μ(Z)) – (n^G ^- n^z)) (2)

when p>q

where R = n_z_ /μ(z) and R_c_ = (n_z_ - n_z_)/(μ(G)-μ(z))

(2) is true when R> R_c_, and otherwise

L(Z)= ( ^n^G /μ(G)) ^n^G ((μ(G)-n_G_ )/μ(G)) ^(μ(G)^- ^n^G) (3)

Next, the solution of $\hat{\text{Z}}$ is found numerically as described in the main text, where $\hat{\text{Z}}$ = {Z: L(Z) $\geq$L(Z′) Z′∈**Z** }.

For making statistical inference on the most likely cluster, Kulldorff calculates the likelihood ratio as follows:

L_0_ is by definition

sup L(Z,p,q) = (n_G_/μ(G)) ^n^G((μ(G)-n_G_ )/μ(G)) ^(μ(G)^- ^n^G) (4)

where p=q

From (1), (2), (3) and (4), the likelihood ratio λ can be written as

λ= L($\hat{\text{Z}}$)/L_0_ (5)

Both the numerator and denominator are functions of the total number of cases, the total number of controls and the number of cases and controls in a “window” (see below) that the scan algorithm defines. In the context of time series, we can call each of these cases or controls “events.” The denominator of this equation depends on the total number of events and not on their temporal distribution along the time series. The likelihood ratio is maximized over a collection of zones **Z**, the total number of which are dependent on the maximum threshold set on the window size (user defined) and the number of events in the times series. The value at which the likelihood ratio statistic is maximized represents the “most likely” cluster. A zone can thus vary in size from as little as one day - if the resolution of the temporal data is a day - to whatever the maximum threshold is defined as by the user. Since the windows move over the time series, anchoring on each day and creating zones of sizes that vary between these extremes, the resulting collection **Z** consists of a multitude of zones. We set the maximum threshold to 30 days. Thus, in theory, the cluster size can vary from 1 to 30 days. Increasing the maximum threshold value did not have an effect on the timing of the cluster or the magnitude of risk detected.

The value of the likelihood ratio test statistic for the “most likely” cluster needs to be compared to a reference distribution, which cannot be theoretically derived. A Monte Carlo simulation , also known as random permutation testing, is used instead. Random replications of the dataset are generated under the null hypothesis conditional on the total number of events n_G_. Unlike a “pure” permutation test, only a certain number of permutations are carried out, because the number of all possible permutations is infinitely large . The likelihood ratio statistic is recalculated for each randomization. Finally, a p value is calculated as rank/(n+1) . The number of randomizations is based on the desired accuracy of the test. If the desired precision for the p value is w, then the number of permutations that need to be calculated can be derived from the binomial variance w = 2, where $\hat{\text{p}}$ is the estimated p value and n is the number of simulations that are to be carried out to achieve ‘w’ precision at that p value. Thus, if we are testing a hypothesis at a p value of 0.05 and a precision of 0.0044 is desired, then a total of 9,814 simulations should be conducted. In the statistical software SaTScan , which we use for our analyses, the number of simulations is restricted to a number ending in “999,” such as 999, 1999, or 9999. Therefore, in our analyses, we do our tests at the 0.05 level and create 9,999 random datasets.

**References**

1. Kulldorff M (1997) A spatial scan statistic. Communications in Statistics - Theory and Methods 26: 1481-1496.

2. Roussas GG (2005) An Introduction to Measure-Theoretic Probability: Gulf Professional Publishing
